# The interplay between attention deficit/hyperactivity disorder and internet addiction: executive dysfunction and insomnia as mediators and the role of physical activity

**DOI:** 10.3389/fpsyt.2026.1737793

**Published:** 2026-02-03

**Authors:** Fangtai Liu, Liping Zhong, Haiyu Chen, Ziwei Teng, Yuhan Su, Jinliang Chen, Yue Qin, Qiong Luo

**Affiliations:** 1 Department of Psychiatry, Brain Hospital of Hunan Province (The Second People’s Hospital of Hunan Province), The School of Clinical Medicine, Hunan University of Chinese Medicine, Changsha, China; 2 College of Physical Education, Hunan University of Technology, Zhuzhou, China; 3 Department of Psychiatry, National Clinical Research Center for Mental Disorders, and National Center for Mental Disorders, The Second Xiangya Hospital of Central South University, Changsha, China; 4 Clinical Psychology Department, Shenzhen Hospital of Integrated Traditional Chinese and Western Medicine, Shenzhen, China; 5 Shenzhen Clinical College of Integrated Chinese and Western Medicine, Guangzhou University of Chinese Medicine, Shenzhen, China

**Keywords:** ADHD, executive dysfunction, insomnia, internet addiction, physical activity

## Abstract

**Background:**

Attention Deficit/Hyperactivity Disorder (ADHD) and internet addiction (IA) are common among college students and often co-exist. This study investigated the relationship between ADHD symptoms, executive dysfunction, insomnia, and IA in Chinese college students.

**Methods:**

A cross-sectional study was conducted in June 2024 at six universities in Hunan Province, China. Demographic data and symptoms of ADHD, IA, executive dysfunction, insomnia, and physical activity were collected via interviews and self-reported questionnaires. Physical activity level was further quantified and categorized using metabolic equivalents (METs) method. Mediation models were performed to explore the path from ADHD to IA and the role of physical activity in IA symptoms.

**Results:**

Among 1925 students, 12.52% had ADHD symptoms, and 14.03% had IA symptoms. ADHD symptoms were related to IA symptoms (total effects: 0.38, p < 0.001; direct effect: 0.111, p = 0.003), mediated by insomnia (0.161, p < 0.001) and executive dysfunction (0.108, p < 0.001). Compared with no physical activity, moderate-level and high-level physical activities were negatively correlated with IA symptoms, with total relative standardized effects of -0.18 (p = 0.001) and -0.42 (p<0.001), respectively. The relative direct effect of high physical activity levels on IA symptoms was -0.29 (p<0.001), regardless of mediation by insomnia (-0.056 (95%CI, -0.094 to -0.021)) and executive dysfunction (-0.067 (95%CI, -0.105 to -0.033)).

**Conclusion:**

ADHD and IA symptoms are prevalent among Chinese college students. Executive dysfunction and insomnia mediate the relationship between ADHD and IA symptoms. Moderate and high-level physical activities were associated with reduced risk of IA symptoms, mediated by executive dysfunction and insomnia. Physical activity may help mitigate IA symptoms in college students.

## 1 Introduction

Attention Deficit/Hyperactivity Disorder (ADHD) is a prevalent neurodevelopmental disorder characterized by developmentally inappropriate levels of inattention, hyperactivity, emotional impulsivity, cognitive deficits, and associated learning difficulties (1). A comprehensive meta-analysis indicates that approximately 6.26% of Chinese children and adolescents meet criteria for ADHD, consistent with estimated global prevalence (2). ADHD symptoms, although typically emerging in childhood, can persist into adulthood. Many adults currently exhibit clinically significant ADHD symptoms, although they did not fully meet diagnostic criteria for ADHD during childhood (3, 4). Adult ADHD causes problems in academic performance, work, and family relationships, leading to increased individual burden and financial pressure on families and society (5, 6). Additionally, individuals with ADHD often have comorbid psychiatric conditions, such as obsessive-compulsive disorder, sleep disturbances, and anxiety disorders (7–9). Consequently, ADHD has raised considerable public health concerns.

Internet addiction (IA) has emerged as a significant public health issue in the digitalized society, characterized by an overwhelming urge to use the internet and an inability to control usage duration (10). Studies indicate ADHD and internet addiction (IA) are closely correlated, and people with ADHD are more likely to develop IA (11, 12). People with IA tend to have poorer mental health and worse self-esteem (13). Teenagers with ADHD symptoms, in particular, may be more likely to develop IA due to poor social skills and increased depressive tendencies. Additionally, IA can reduce sleep quality by shortening sleep duration (14). According to research by Li and his colleagues, people with ADHD may be more susceptible to developing an addiction to online activities due to their impulsivity and concentration problems (15). Furthermore, a study conducted by Chou and his colleagues revealed that for individuals with ADHD, social challenges may prompt the use of online interactions as a compensatory strategy, thereby increasing the risk for IA (16). Demirtaş and colleagues found that people with ADHD often experience mental health issues like anxiety and depression, which are closely related to IA (17). People showing signs of depression might use online activities as a way to escape from reality, thereby worsening their IA (18). Ko et al. noted that individuals with ADHD in urban areas may face higher level of stress and less social support, making them more vulnerable to developing an IA. This situation is particularly relevant in the context of Chinese universities (19). As the country with the world’s largest online population, China has developed a highly integrated digital environment. Widely used platforms such as Honor of Kings, WeChat, and Douyin are deeply woven into everyday life, which may increase users’ vulnerability to internet addiction (20, 21). Despite substantial evidence demonstrating the frequent co-occurrence of ADHD and IA, the precise directional relationship between these conditions remains unclear.

Executive dysfunction, a common comorbidity of ADHD, affects domains such as working memory, planning, attentional control, and inhibitory control, which are prevalent among individuals with ADHD (22). A long-term study suggests that these issues with executive function, especially problems with impulse control and self-regulation, may put people with ADHD at a higher risk for IA (23). Furthermore, insomnia, commonly seen in ADHD patients, may exacerbate their executive function challenges (24, 25). However, current studies typically focused on these factors separately, with limited consideration of their interplay, thus leaving their complex interactions underexplored.

Physical activity, as a non-pharmacological intervention, has been shown to effectively reduce symptoms of IA and improve related mental health outcomes in randomized controlled trials (26). Additionally, physical activity may also mitigate deficits in executive functioning and alleviate insomnia (27). However, its role as a potential factor in preventing IA risk has not been quantitatively evaluated.

Building on this foundation, we hypothesized that ADHD symptoms, executive dysfunction, and insomnia shared an intrinsic link with IA symptoms and that physical activity was correlated with these conditions. Firstly, we investigated the occurrence of ADHD and IA symptoms among Chinese college students through a cross-sectional study. Then, we evaluated and refined our proposed framework linking ADHD symptoms, executive dysfunction, insomnia, and IA symptoms, by using structural equation modeling (SEM). Finally, we explored the role of physical activity in these relationships. We aimed to explore the mediating effects of executive dysfunction and insomnia between ADHD and IA symptoms and the role of different intensities of physical activity on IA.

## 2 Materials and methods

### 2.1 Participants

A cross-sectional study was undertaken among students sampled from six scientific and technology universities in Hunan Province, China. Individuals were included if they (a) were currently enrolled university students aged 16 years or older, (b) demonstrated the cognitive and linguistic capacity to complete the assessment, and (c) were willing to provide documented informed consent. Of the 2,188 students approached, 263 were excluded for not completing all questions. As a result, 1,925 students were included in the study.

### 2.2 Procedures

All participants received the surveys from their teachers in June 2024. Clear instructions were provided throughout the questionnaire. Teachers received professional instruction from experienced psychiatric specialists to help participants understand each question’s purpose and content, and they offered crucial assistance during survey completion to clarify any misunderstandings or uncertainties. All participants provided informed consent, and participation was entirely voluntary. The study was approved by the Ethics Committee of the Brain Hospital of Hunan Province (2024K008), and all procedures were carried out in accordance with the ethical principles outlined in the Declaration of Helsinki. To protect privacy and encourage honest responses, the survey was conducted anonymously.

### 2.3 Clinical measures

Demographic information

Basic demographic data collected included the students’ age, sex, and their grades.

#### 2.3.1 Adult ADHD SELF-REPORT SCALE

The ASRS comprises 18 items, each with five possible responses: never, rarely, occasionally, often, and very often. This study used a binary scoring method suggested by Kessler et al., where each question was scored as either 0 or 1 based on symptom severity (28). The scale has two subscales—nine for inattention and nine for hyperactivity—each with possible scores ranging from 0 to 9. A score of 1 is given if the answer indicates clinically significant symptoms; otherwise, it is scored as 0. Seven questions have clinical relevance when answered as sometimes, often, or very often, while the other 11 require answers of frequently or very frequently to be clinically relevant. Based on DSM-5 criteria, this scale yields high specificity in identifying adult ADHD symptoms and demonstrates acceptable internal consistency, with reported Cronbach’s α between 0.63 and 0.72 (29). In this study, the ASRS was used as an ADHD screening tool, with a total score greater than nine indicating clinical level ADHD symptoms, consistent with previous studies (30).

#### 2.3.2 Barkley Deficits in executive functioning scale, short form

The BDEFS-SF is a shortened version of the 89-item BDEFS, consisting of 20 items that measure executive dysfunction through self-report (31). Each item uses a four-point Likert scale (1–4) based on how often symptoms occur: never or seldom, sometimes, often, and always. Scores range from 18 to 72, with higher scores indicating more severe executive function (EF) problems (32). The BDEFS-SF has a Cronbach’s alpha coefficient of 0.94, indicating excellent internal consistency (32).

#### 2.3.3 Athens insomnia scale

The AIS-8 is an eight-item self-assessment tool designed to evaluate sleep issues according to ICD-10 criteria. The first five items address sleep induction, nighttime awakenings, final awakenings, overall sleep duration, and sleep quality, while the final three items assess functioning capacity, well-being, and daytime sleepiness (33). The AIS-8 is a four-point Likert scale and has a total score ranging from 0 to 24. This scale demonstrates high internal consistency with a Cronbach’s α of 0.89, and a cutoff score of 6, suggesting an elevated risk of insomnia.

#### 2.3.4 Chinese internet addiction scale-revision

The CIAS-R is a 19-item self-report questionnaire that evaluates IA symptoms across four categories: tolerance, compulsive use and withdrawal, interpersonal and health-related issues, and time management concerns (34). Each item is rated on a four-point Likert scale, yielding a total score ranging from 19 to 76. A score above 53 is indicative of clinically significant IA symptoms. The CIAS-R has shown strong validity and reliability in China, with a Cronbach’s alpha of 0.96 (34).

#### 2.3.5 Metabolic equivalents

Metabolic equivalent (MET) is a common method to express physical activity levels as multiples of resting metabolic rate (RMR) (35). Our survey included physical activity-related questions, covering frequency, duration, and types of activities. MET values were calculated based on participant responses using the method described by Pearce et al. (36) and converted into persistent marginal MET-hours (mMET-h). We then categorized participants into low, medium, and high physical activity groups using quartiles and compared them against a group that did not engage in regular physical exercise.

### 2.4 Statistics

The Kolmogorov–Smirnov test was used to assess normality of continuous variables. All rating scales were non-normally distributed. The χ2 test was then utilized for categorical data, while the Mann-Whitney U test was utilized to investigate differences in quantitative variables. A structural equation model (SEM) was employed to evaluate the mediating effects of executive dysfunction and insomnia on the association between ADHD and IA symptoms. Spearman correlations were used as the basis for SEM. To be considered acceptable, the SEM had to meet the following criteria: Tucker–Lewis’s index (TLI) > 0.95, standardized root mean square residual (SRMR) < 0.08, comparative fit index (CFI) > 0.95, and root mean square error of approximation (RMSEA) < 0.08 (37, 38). All analyses were conducted using R. Studio and SPSS 26.0 (IBM, Inc., Chicago), with a two-tailed significance level of 0.05.

## 3 Results

### 3.1 Demographic and clinical characteristics

After excluding 263 of the 2,188 college students who participated in the survey, the response rate was 87.98%. **Table 1** presented the sociodemographic and clinical characteristics of the participants, including 703 (36.5%) females and 1,222 (63.5%) males. Among them, 14.03% (270 out of 1,925) were identified as having IA symptoms, and 12.52% (241 out of 1925) had ADHD symptoms. The sociodemographic and clinical traits of students with and without IA symptoms was shown in **Table 1**. Univariate logistic regression was used for all variables, with the χ2 test applied to categorical data and the Mann-Whitney U test for quantitative variables. No statistically significant differences were observed in age, sex, or grade level between the two groups (all p > 0.05).

**Table 1:** Demographics and clinical symptoms between participants with and without internet addiction.

Variables		Internet addiction		
	Overall	No	Yes	p-value
	(N = 1925)	(N = 1655)	(N = 270)	
	N (%)	N (%)	N (%)	
Sex				
male	1222 (63.5)	1047 (63.3)	175 (64.8)	0.634^a^
female	703 (36.5)	608 (36.7)	95 (35.2)	
Grade				
freshman	1161 (60.3)	992 (59.9)	169 (62.6)	0.233^a^
sophomore	446 (23.2)	379 (22.9)	67 (24.8)	
junior	299 (15.5)	268 (16.2)	31 (11.5)	
senior	19 (1.0)	16 (1)	3 (1.1)	
Exercise habit				0.027^a^
No	771 (40.1)	646 (39)	125 (46.3)	
Yes	1154 (59.9)	1009 (61)	145 (53.7)	
Exercise type				
None	771 (40.1)	646 (39)	125 (46.3)	0.058^a^
High intensity, MET	513 (26.6)	449 (27.1)	64 (23.7)	
Medium intensity, MET	370 (19.2)	321 (19.4)	49 (18.1)	
Medium-high intensity, MET	7 (0.4)	5 (0.3)	2 (0.7)	
Low intensity, MET	264 (13.7)	234 (14.1)	264 (13.7)	
		Mean (SD)	Mean (SD)	
Age, year	19.4 (1.20)	19.4 (1.21)	19.37 (1.11)	0.672^b^
Exercise frequency, times/week	1.5 (1.64)	1.52 (1.64)	1.37 (1.64)	0.065^b^
Time per exercise, min	33.39 (37.2)	34.11 (37.48)	29 (35.17)	0.026^*b^
Time of adherence to exercise, month	5.59 (16.4)	5.81 (17.14)	4.22 (10.95)	0.006^*b^
ASRS total score	4.68 (3.23)	4.29 (3)	7.06 (3.54)	<0.001^***b^
AIS total score	4.74 (3.71)	4.21 (3.3)	7.97 (4.38)	<0.001^***b^
BDEFS-SF total score	38.06 (12.01)	36.58 (11.11)	47.15 (13.29)	<0.001^***b^

MET, metabolic equivalent; ASRS, Adult ADHD Self-Report Scale; AIS, Athens Insomnia Scale; BDEFS-SF, Barkley Deficits in Executive Functioning Scale, Short Form.

Statistic methods: a, χ^2^ test. b, Mann-Whitney test. *p < 0.05. **p < 0.01. ***p < 0.001.

Students with IA symptoms had lower percentages of exercise habits, engaged in lower intensity exercise, spent less time per exercise session, and showed poorer adherence to exercise routines compared to those without IA symptoms (p < 0.05). Additionally, their scores were significantly higher on the BDEFS-SF, AIS, and overall ASRS (all p < 0.001; **Table 1**). Even after adjusting for factors like age, sex, and grade level, multiple linear regression analysis showed that exercise behaviors, insomnia, executive dysfunction, and ADHD symptoms were strongly linked to IA symptoms (all p < 0.05; **Table 2**).

**Table 2:** Multiple linear regression analysis for variables correlated with internet addiction.

Variables	Unstandardized coefficient, B	Standardized coefficient, Beta	t	p- value	95 CI LL	95 CI UL
Exercise habits	-2.004	-0.09	-4.53	<0.001	-2.871	-1.136
AIS total scores	0.883	0.301	13.731	<0.001	0.757	1.009
ASRS total scores	0.39	0.115	5.173	<0.001	0.242	0.537
BDEF-SF total scores	0.205	0.226	9.762	<0.001	0.164	0.246

The model was adjusted for sex, age, and grade.

AIS, Athens Insomnia Scale; ASRS, Adult ADHD Self-Report Scale; BDEFS-SF, Barkley Deficits in Executive Functioning Scale, Short Form.

### 3.2 Pathways from ADHD symptoms to IA symptoms

**Figure 1:**
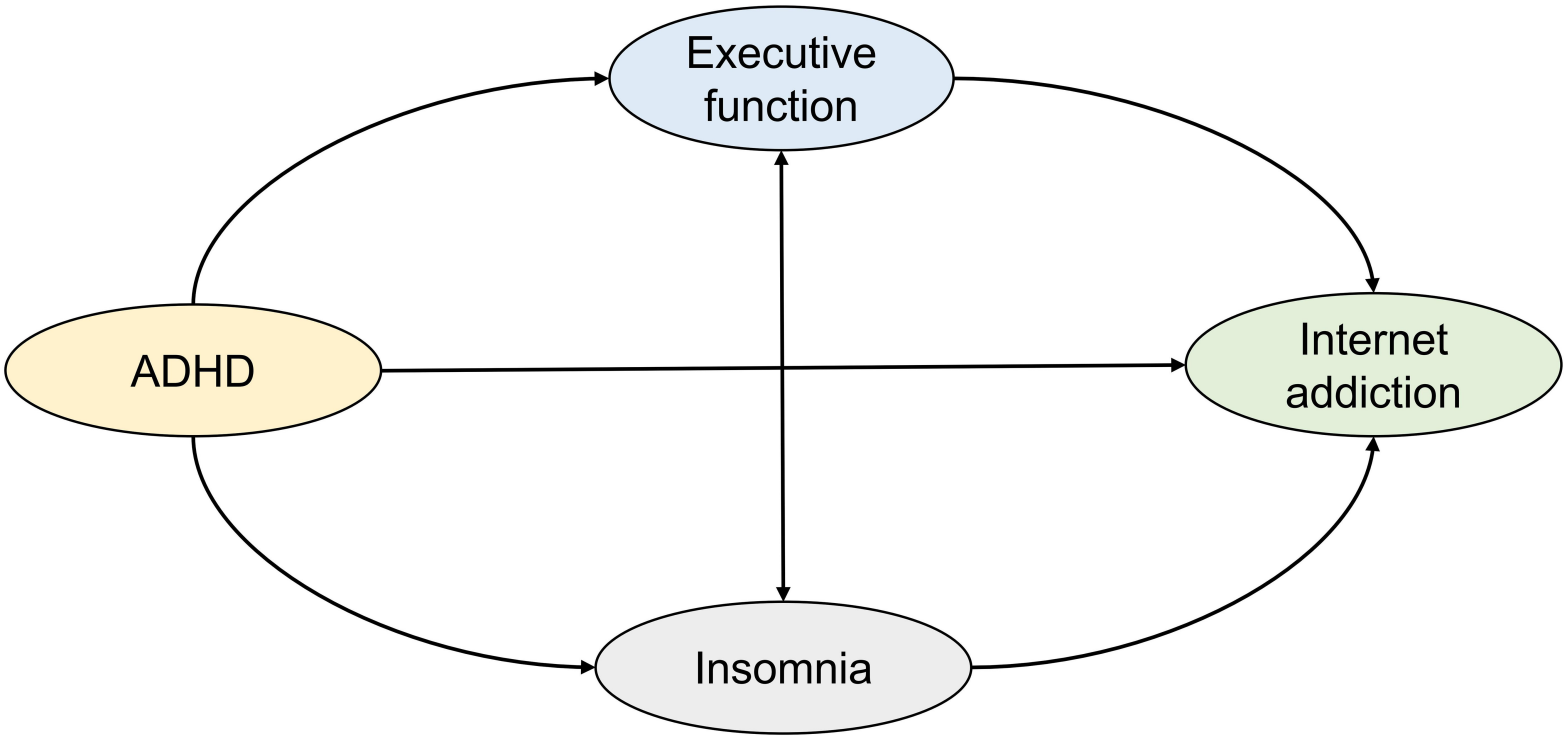
The hypothesized model for the association between ADHD and internet addiction. Abbreviations: ADHD, Attention Deficit/Hyperactivity Disorder.

**Figure 2:**
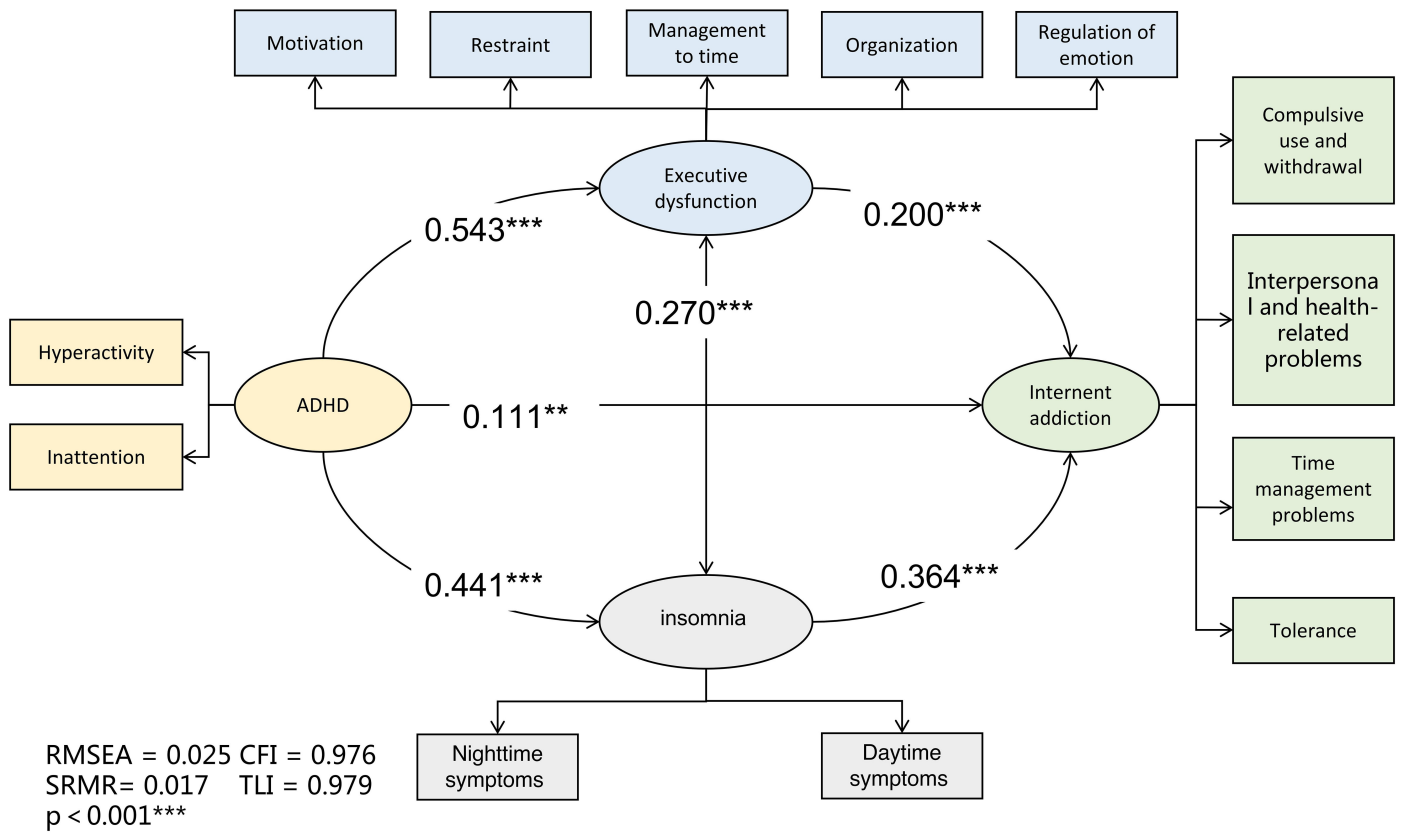
The SEM for the association between internet addiction and ADHD mediated by insomnia and executive dysfunction. Abbreviations: ADHD, Attention Deficit/Hyperactivity Disorder. *p < 0.05. **p < 0.01. ***p < 0.001.

There were positive correlations between all symptoms and sub-symptoms, including executive dysfunction, insomnia, and ADHD symptoms (all p < 0.01; **Table 3**). The absence of multicollinearity among these factors supported the validity of the subsequent mediation analysis. Model fit indices indicated that the proposed model (**Figure 1**) fit the data well. The path model (**Figure 2**) showed a total standardized effect of 0.38 (p < 0.001), indicating a positive correlation between IA and ADHD symptoms.

**Table 3:** Spearman rank correlations between internet addiction, insomnia, executive dysfunction, and ADHD.

Rho	CIAS-R	CIAS-R-Compulsive use and withdrawal	CIAS-R-Tolerance	CIAS-R-Time management problems	CIAS-R-Interpersonal and health-related problems
AIS total scores	0.449^**^	0.405^**^	0.389^**^	0.409^**^	0.436^**^
AIS-Nighttime symptoms	0.395^**^	0.357^**^	0.335^**^	0.371^**^	0.383^**^
AIS-Daytime symptoms	0.432^**^	0.390^**^	0.387^**^	0.376^**^	0.424^**^
ASRS total scores	0.338^**^	0.305^**^	0.301^**^	0.304^**^	0.324^**^
Inattention	0.293^**^	0.259^**^	0.265^**^	0.259^**^	0.287^**^
Hyperactivity	0.305^**^	0.283^**^	0.268^**^	0.283^**^	0.285^**^
BDEF-SF total scores	0.386^**^	0.353^**^	0.342^**^	0.359^**^	0.361^**^
BDEF-SF-Motivation	0.376^**^	0.345^**^	0.324^**^	0.350^**^	0.356^**^
BDEF-SF-Restraint	0.316^**^	0.295^**^	0.280^**^	0.301^**^	0.286^**^
BDEF-SF-Management to time	0.381^**^	0.348^**^	0.351^**^	0.345^**^	0.355^**^
BDEF-SF-Organization	0.354^**^	0.328^**^	0.314^**^	0.315^**^	0.339^**^
BDEF-SF-Regulation of emotion	0.355^**^	0.328^**^	0.308^**^	0.333^**^	0.331^**^

CIAS-R, Chinese Internet Addiction Scale-Revision; AIS, Athens Insomnia Scale; ASRS, Adult ADHD Self-Report Scale; BDEFS-SF, Barkley Deficits in Executive Functioning Scale, Short Form. ** p< 0.01.

IA symptoms were directly associated with executive dysfunction, insomnia, and ADHD symptoms, with significant covariation between executive dysfunction and insomnia (all p < 0.001). Specifically, ADHD symptoms had an impact on IA symptoms, with a standardized direct effect of 0.111(p=0.003) and a standardized indirect effect of 0.269 (p < 0.001). The path coefficient measuring the impact of ADHD symptoms on IA symptoms through insomnia was 0.161 (p < 0.001), while executive dysfunction had a coefficient of 0.108 (p < 0.001), indicating that they mediated the effect of ADHD symptoms on IA symptoms.

### 3.3 Pathways from physical activity to IA symptoms

**Figure 3:**
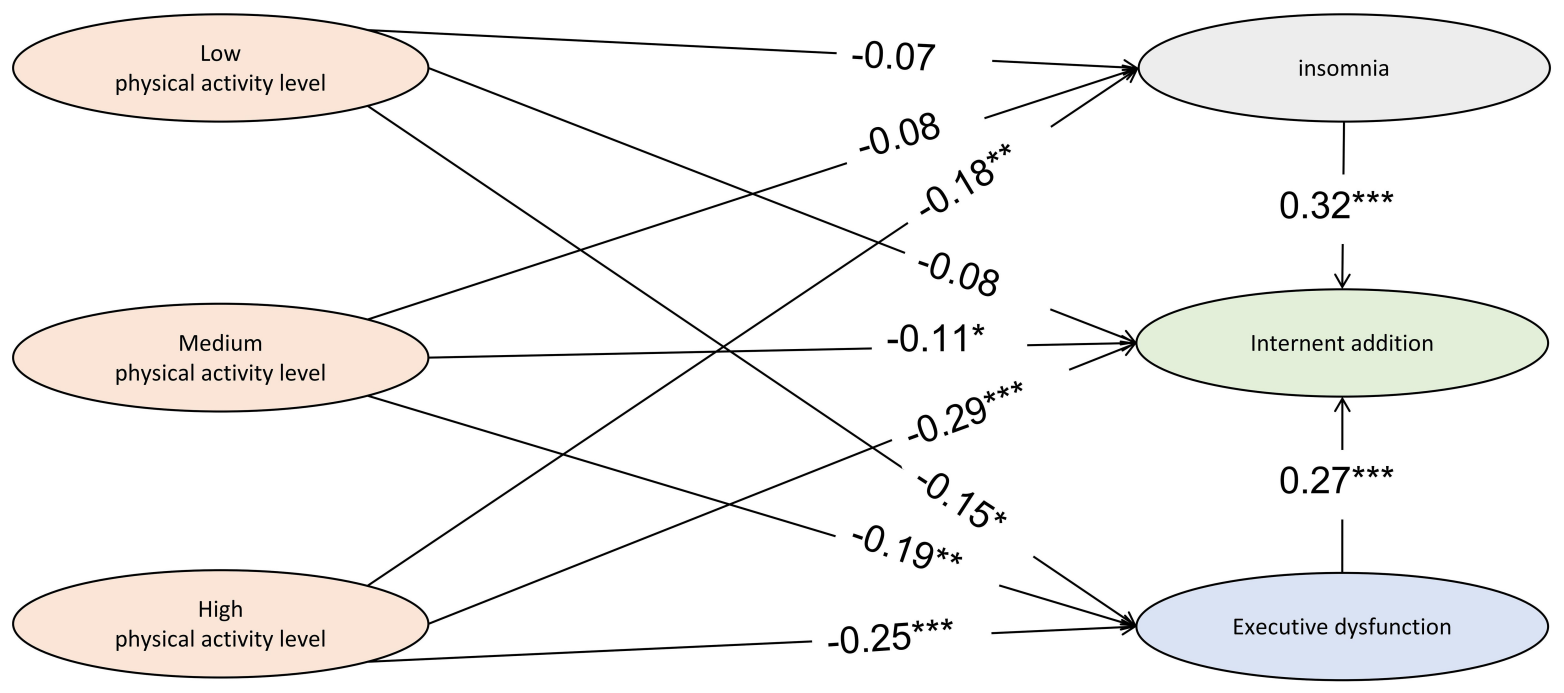
The association between internet addiction and physical activity level mediated by insomnia and executive dysfunction. *p < 0.05. **p < 0.01. ***p < 0.001.

**Supplementary Table S1** showed the sociodemographic and clinical traits of students with and without exercise habits. Students with exercise habits had lower total scores on the CIAS-R, AIS, and BDEFS-SF (p < 0.001), were typically in lower grade levels, and were more likely to be male than those without exercise habits. Moderate physical activity levels were negatively associated with IA symptoms, according to the simple mediation analysis model (**Figure 3**; **Supplementary Table S2**), with a relative direct standardized effect of -0.11 (p = 0.033) and a relative total standardized effect of -0.18 (p = 0.001) compared to those who did not exercise. The path coefficient from moderate physical activity level to IA symptoms mediated by executive function was -0.052 (95% CI, -0.085 to -0.0197). High levels of physical activity were even more strongly inversely correlated with IA symptoms, with a relative direct standardized effect of -0.29 (p < 0.001) and a relative total standardized effect of -0.42 (p < 0.001) when compared to those who did not exercise regularly. The partial relative mediating effect of insomnia on the path from high levels of physical activity to IA symptoms was -0.056 (95% CI, -0.094 to -0.021), which was -0.067 (95% CI, -0.105 to -0.033) for executive dysfunction. However, low amounts of exercise had no significant impact on IA symptoms when compared to no activity.

## 4 Discussion

In this study, we assessed the prevalence of ADHD and internet addiction (IA) symptoms among Chinese college students, revealing rates of 12.52% for ADHD symptoms and 14.03% for IA symptoms. Findings of structural equation modeling (SEM) demonstrated that executive dysfunction and insomnia jointly mediated the relationship between ADHD and IA symptoms. Specifically, physical activity levels were inversely associated with IA symptoms, with this relationship also being mediated by executive dysfunction and insomnia. These results suggest that executive dysfunction and insomnia serve as key mediators in the pathway from ADHD symptoms to IA, and that physical activity may prevent IA risk by affecting these mediators.

A 2020 meta-analysis reported a global prevalence of 2.58% for persistent adult ADHD and 6.76% for symptomatic adult ADHD (39). Previous studies reported varying prevalence rates of ADHD among Chinese college students. One study of 8,098 students reported a prevalence of 7.26%, whereas another involving 5,693 students found 3.5% (40, 41). The prevalence of ADHD symptoms in our sample was 1.7 to 3.6 times higher than those reported in previous studies. This discrepancy may be attributed to variations in sample size and the scoring method employed in the ASRS.

Research indicates that the prevalence of IA among Chinese university students reaches 7.7% (42). Epidemiological evidence also indicates a high prevalence of IA within the general Chinese population, especially among adolescents (43). This suggests that IA symptoms may occur in individuals without pre-existing conditions, potentially explaining the high detection rate of IA symptoms among college students in our study. Another study involving 8,098 Chinese college students revealed that the prevalence rates of IA were 7.21% in males and 8.17% in females (40). In our study, the detection rate of IA symptoms among college students was found to be relatively high, with a prevalence rate of 14.03%. A South Korean study investigating the relationship between ADHD and IA suggested that IA might be more closely associated with the cognitive and behavioral symptoms of ADHD rather than ADHD diagnosis, and the comorbidity between ADHD and IA might be linked to functional and structural brain abnormalities associated with excessive and pathological internet use (44). These findings collectively indicate that the related dysfunctions in ADHD symptoms are highly likely to lead to disordered internet use.

Effective interventions for individuals with ADHD symptoms could help them in managing their internet use. Our SEM analysis revealed that executive dysfunction serves as a mediator in the relationship between ADHD and IA symptoms. Symptoms of ADHD can lead to difficulties in planning, organization, and time management, which are characteristics of executive dysfunction (45). This dysfunction may impair self-regulation and task management, leading individuals to engage in internet activities as an escape from real-world challenges (46). Therefore, executive dysfunction stemming from ADHD symptoms may increase reliance on the internet, supporting our findings and confirming its mediating role in the ADHD-IA symptoms relationship. Notably, moderate online gaming has been shown to enhance executive function in college students, while excessive use at subclinical levels may similarly impair it (47). Consequently, clinical interventions should adopt a differentiated perspective that acknowledges and accounts for the potential compensatory or functional benefits of internet use.

Another potential mediating factor observed in this study was insomnia. A study focusing on pediatric ADHD patients found that sleep disturbances like insomnia are frequently observed comorbidities in this population (48). Furthermore, Grant et al. reported that insomnia may indirectly contribute to the onset and progression of IA by impairing emotional regulation and impulse control (49). Convergent evidence from the literature and our study confirms that insomnia is a significant mediator in the pathway from ADHD symptoms to IA.

Moreover, this study found that high-level physical activity was negatively associated with IA symptoms, which were mediated by insomnia and executive dysfunction. These findings align with previous research. For instance, A 2015 meta-analysis identified physical activity functions as a comprehensive intervention that directly reduces the risk of IA while also providing indirect protection through the enhancement of mental health (50). Additionally, a systematic review by Alimoradi et al. reported a significant association between IA and sleep disturbances and noted that physical activity improves sleep quality, thereby indirectly reducing IA risk (51). Other studies further suggest that physical activity may alleviate IA symptoms and reduce its incidence (52–54). Notably, our study identified that moderate-to-high levels of physical activity were associated with a reduced risk of IA, thereby offering a supplement to previous findings in the field. However a 2023 multinational study found that among university students in Portugal and Poland, those with a history of SARS-CoV-2 infection, particularly in cases of recurrent infection, exhibited an elevated risk of internet addiction, when regularly engaging in physical activity (55). We speculated that this phenomenon may be associated with regional factors and the impact of COVID-19.

The findings of this study yielded significant clinical and public health insights. First, this study established the prevalence of internet addiction among Chinese college students through a large-scale survey. Second, the application of validated psychometric instruments and SEM allowed for the empirical validation of a multidimensional interaction model that incorporates ADHD symptoms, executive dysfunction, insomnia, and IA. The analysis confirmed the direct effect of ADHD symptoms on IA and identified an indirect pathway mediated by executive dysfunction and insomnia. It also revealed that physical activity intensity moderated the risk of IA. Third, the findings highlighted the need to address both neuropsychological factors, such as executive function and sleep disturbances, and modifiable lifestyle factors, such as physical activity of appropriate intensity, in the design of intervention strategies. This cross-sectional evidence offers preliminary insights that could inform the development of integrated intervention frameworks aimed at reducing IA risk among college students.

The current study has several limitations. First, using self-reported scales may lead to recall bias and reporting bias. Second, the sample was recruited from six universities in Hunan Province, limiting the generalizability of the findings. Third, the cross-sectional design could not determine the causal relationships among these variables. Finally, potentially relevant confounders like mood symptoms or medication were not considered in this study. Future longitudinal studies should incorporate other comorbid psychopathological conditions as confounders and elucidate possible bidirectional and dynamic interplay between ADHD symptoms and IA.

## 5 Conclusion

This study demonstrates that IA symptoms are highly prevalent among Chinese college students and are associated with ADHD symptoms. Furthermore, our findings highlight the mediating roles of executive dysfunction and insomnia in the relationship between ADHD symptoms and IA symptoms. These results underscore the importance of addressing these modifiable factors in clinical and educational settings. Caregivers and healthcare providers should consider integrating assessments of executive functioning, sleep quality, and physical activity into interventions for students with IA symptoms to optimize outcomes.

## Data Availability

The raw data supporting the conclusions of this article will be made available by the authors, without undue reservation.
