# Supplementary material for: The interplay between attention deficit/hyperactivity disorder and internet addiction: executive dysfunction and insomnia as mediators and the role of physical activity
Source: Front Psychiatry. 2026 Feb 3;17:1737793. doi: 10.3389/fpsyt.2026.1737793 (PMC12909545; doi:10.3389/fpsyt.2026.1737793)
Supplement: Supplementary file 1 [file Table1.docx]

Table S1. Demographics and clinical symptoms between participants with and without exercise habits.

|  |  | Exercise habits | |  |
| --- | --- | --- | --- | --- |
|  | Overall | No | Yes | p-value |
|  | (N=1925) | (N=771) | (N=1154) |  |
|  | N (%) | N (%) | N (%) |  |
| sex |  |  |  |  |
| male | 1222 (63.5) | 432 (56.0) | 790 (68.5) | 0.001^**a^ |
| female | 703 (36.5) | 339 (44.0) | 364 (31.5) |  |
| grade |  |  |  |  |
| freshman | 1161 (60.3) | 445 (57.7) | 716 (62.0) | 0.001^**a^ |
| sophomore | 446 (23.2) | 170 (22.0) | 276 (23.9) |  |
| junior | 299 (15.5) | 142 (18.4) | 157 (13.6) |  |
| senior | 19 (1.0) | 14 (1.8) | 5 (0.4) |  |
| Exercise type |  |  |  |  |
| None | 771 (40.1) | 771 (100) | 0 (0) | 0.001^**a^ |
| High intensity, MET | 513 (26.6) | 0 (0) | 513 (44.5) |  |
| Medium intensity, MET | 370 (19.2) | 0 (0) | 370 (32.1) |  |
| Medium-high intensity, MET | 7 (0.4) | 0 (0) | 7 (0.6) |  |
| Low intensity, MET | 264 (13.7) | 0 (0) | 264 (22.9) |  |
|  |  | Mean (SD) | Mean (SD) |  |
| Age, year | 19.4 (1.20) | 19.4 (1.26) | 19.4 (1.15) | 0.506^b^ |
| Exercise frequency, times/ week | 1.5 (1.64) | 0 (0) | 2.5 (1.41) | <0.001^***b^ |
| Time per exercise, min | 33.39 (37.2) | 0 (0) | 55.7 (32.65) | <0.001^***b^ |
| Time of adherence to exercise, month | 5.59 (16.4) | 0 (0) | 9.33 (20.4) | <0.001^***b^ |
| ASRS-9 total score | 4.68 (3.23) | 4.70 (3.22) | 4.67 (3.23) | 0.798^b^ |
| CIAS-R total score | 41.7 (10.9) | 43.4 (10.6) | 40.6 (10.9) | 0.001^**b^ |
| AIS total score | 4.74 (3.71) | 5.08 (3.79) | 4.51 (3.64) | 0.001^**b^ |
| BDEFS-SF total score | 38.06 (12.01) | 39.49 (11.87) | 37.11 (12.02) | <0.001^***b^ |

Abbreviations: MET, metablic equivalent; ASRS-9, Adult ADHD Self-Report Scale; AIS, Athens Insomnia Scale; BDEFS-SF, Barkley Deficits in Executive Functioning Scale, Short Form.

Statistic methods: a, χ^2^ test. b, Mann-Whitney test. *p < 0.05. **p < 0.01. ***p < 0.001.

Table S2. the relative total, direct, and indirect effects of sports on internet addiction.

|  | Effect | t | p-value | LLCI | ULCI | Omnibus test p-value |
| --- | --- | --- | --- | --- | --- | --- |
| Relative total effects |  |  |  |  |  | <0.001 |
| Low physical activity level  ->Internent addition | -1.5696 | -1.8674 | .0620 | -3.2180 | .0789 |  |
| Medium physical activity level  ->Internent addition | -2.0098 | -3.1822 | .0015 | -3.2484 | -.7712 |  |
| High physical activity level  ->Internent addition | -4.5339 | -7.0142 | .0000 | -5.8017 | -3.266 |  |
| Relative direct effects |  |  |  |  |  | <0.001 |
| Low physical activity level  ->Internent addition | -.8700 | -1.1904 | .2340 | -2.3034 | .5633 |  |
| Medium physical activity level  ->Internent addition | -1.1765 | .5501 | .0326 | -2.2554 | -.0975 |  |
| High physical activity level  ->Internent addition | -3.1895 | -5.6522 | .0000 | -4.2962 | -2.0828 |  |
|  | Effect | BootLLCI | BootULCI | Standardized Effect | Standardized BootLLCI | Standardized BootULCI |
| Relative indirect effects |  |  |  |  |  |  |
| Low physical activity level  ->insomnia ->Internent addition | -.2519 | -.7717 | .2566 | -.0231 | -.0710 | .0234 |
| Medium physical activity level  ->insomnia ->Internent addition | -.2730 | -.6843 | .1458 | -.0251 | -.0629 | .0134 |
| High physical activity level  ->insomnia ->Internent addition | -.6183 | -1.0240 | -.2267 | -.0568 | -.0941 | -.0209 |
| Low physical activity level  ->Executive dysfunction  ->Internent addition | -.4476 | -.8951 | -.0292 | -.0411 | -.0817 | -.0026 |
| Medium physical activity level  ->Executive dysfunction  ->Internent addition | -.5603 | -.9275 | -.2143 | -.0515 | -.0850 | -.0197 |
| High physical activity level  ->Executive dysfunction  ->Internent addition | -.7262 | -1.1492 | -.3550 | -.0667 | -.1048 | -.0327 |
